# The Isolation and Characterization of a Novel Psychrotolerant Cellulolytic Bacterium, *Microbacterium* sp. QXD-8^T^

**DOI:** 10.3390/microorganisms12020303

**Published:** 2024-01-31

**Authors:** Peng An, Changjialian Yang, Wei Li, Dahe Zhao, Hua Xiang

**Affiliations:** 1 College of Life Science, Sichuan Normal University, Chengdu 610101, China; 17308148085@163.com (P.A.); liwei001@sicnu.edu.cn (W.L.); 2 State Key Laboratory of Microbial Resources, Institute of Microbiology, Chinese Academy of Sciences, Beijing 100101, China; yangchangjialian22@mails.ucas.ac.cn; 3 University of Chinese Academy of Sciences, Beijing 100049, China

**Keywords:** polyphasic taxonomy, cellulose degradation, functional genomics, *Microbacterium psychrotolerans*

## Abstract

Cellulolytic microorganisms play a crucial role in agricultural waste disposal. Strain QXD-8^T^ was isolated from soil in northern China. Similarity analyses of the 16S rRNA gene, as well as the 120 conserved genes in the whole-genome sequence, indicate that it represents a novel species within the genus *Microbacterium*. The *Microbacterium* sp. QXD-8^T^ was able to grow on the CAM plate with sodium carboxymethyl cellulose as a carbon source at 15 °C, forming a transparent hydrolysis circle after Congo red staining, even though the optimal temperature for the growth and cellulose degradation of strain QXD-8^T^ was 28 °C. In the liquid medium, it effectively degraded cellulose and produced reducing sugars. Functional annotation revealed the presence of encoding genes for the GH5, GH6, and GH10 enzyme families with endoglucanase activity, as well as the GH1, GH3, GH39, and GH116 enzyme families with *β*-glucosidase activity. Additionally, two proteins in the GH6 family, one in the GH10, and two of nine proteins in the GH3 were predicted to contain a signal peptide and transmembrane region, suggesting their potential for extracellularly degrade cellulose. Based on the physiological features of the type strain QXD-8^T^, we propose the name *Microbacterium psychrotolerans* for this novel species. This study expands the diversity of psychrotolerant cellulolytic bacteria and provides a potential microbial resource for straw returning in high-latitude areas at low temperatures.

## 1. Introduction

Cellulose is the most widely distributed and abundant carbohydrate polymer in nature [1]. It is composed of D-glucose linked by *β*-1,4-glycosidic bonds. Natural cellulose forms high crystallinity through intermolecular and intramolecular hydrogen bonds, leading to high resistance to enzymatic hydrolysis [2]. The crystallinity depends on the proportions of crystalline and amorphous areas in the cellulose materials [3]. Cellulose is hydrolyzed by cellulases, which can be divided into three types according to their catalytic modes [4]: (i) endo-*β*-1,4-glucanase (EC 3.2.1.4); (ii) exoglucanase, including 1,4-*β*-D-glucan glucanohydrolases (glucanohydrolase) (EC 3.2.1.74) and cellobiohydrolase (EC 3.2.1.91); and (iii) *β*-glucosidases (EC 3.2.1.21) [3,5]. Endoglucanase randomly cuts at the internal amorphous sites of the cellulosic polysaccharide chain to produce oligosaccharides of different lengths. After that, new ends form. In contrast, exoglucanase functions at the reducing or nonreducing ends of the cellulosic polysaccharide chain, and then releases glucose (using glucanohydrolase) or cellobiose (using cellobiohydrolase) as the main products. *β*-glucosidase hydrolyzes soluble cellodextrin and cellobiose to glucose [2,6]. All three types can exist and function inside or outside cells [7]. In total, there are 26 known hydrolase families that exhibit cellulolytic activity: endoglucanase (GH5, GH6, GH7, GH8, GH9, GH10, GH12, GH44, GH45, GH48, GH51, GH74, GH124, GH131, and GH148), *β*-glucosidase (GH1, GH2, GH3, GH30, GH39, and GH116), cellulase/cellulprotamine phosphorylase (GH94), and hydrolyzing cellulose monooxygenase (AA9, AA10, AA15, and AA16) [8]. These enzyme families are key to predicting the functional genes of a cellulolytic microbe.

Most studies of cellulolytic bacteria have focused on mesophilic or thermophilic representatives in compost. Typically, these microorganisms exhibit optimum cellulase activity at room temperature or at high temperatures. Mayende et al. obtained samples from lucerne and a garden compost heap (50 °C). The samples LM01 and LM04 showed the highest cellulase activity at 60 °C, while samples LM02 and LM03 exhibited the highest cellulase activity at 70 °C, indicating thermophilic cellulase activity [9]. Legodi et al. isolated ten cellulose-degrading strains of bacteria from decomposed banana agro-waste and decomposed *Strelitzia alba* plant, which had high enzyme activity at 30–40 °C [10]. Generally, low-temperature-resistant and cellulose-degrading microorganisms can maintain considerable activity [11]. In particular, low-temperature-resistant microorganisms can expedite the process of composting [12]. Such an example is that the gene M6A encoding a novel cold-adapted endoglucanase was cloned from *Microbacterium kitamiense* Sa12, a strain isolated from a wasteland in Saga, Qinghai-Tibetan Plateau. Compared with an optimum temperature of 35 °C, the relative activity of the enzyme was 30–40% at 0–5 °C [13].

The genus *Microbacterium* was established based on the type species *Microbacterium lacticum*, which was isolated in a study using lactic acid bacteria [14]. Later, the genus *Aureobacterium*, classified in 1983 [15], was re-emended into the genus *Microbacterium* [16]. As of November 2023, the genus *Microbacterium* comprises a total of 160 validly published species (https://lpsn.dsmz.de/genus/microbacterium, accessed on 23 November 2023), and these species are Gram-stain-positive and rod-shaped, but are not able to form spore [17]. Members of the genus *Microbacterium* have been isolated from a diverse array of environments, including soil, sediment [18], activated sludge [19], insects [20], fresh water [21], marine environments [22], plants [23], and human clinical specimens [24]. At present, it has been reported that there is a significant correlation between *Microbacterium* and hemicellulose digestion in the intestinal tract of locusts [25].

In this study, we present a novel species of the genus *Microbacterium* and describe the phenotypic and genotypic properties of this new strain with the ability of degrading cellulose under a wide range of temperatures and releasing reducing sugar at low temperatures. Furthermore, we elucidated the genetic basis for metabolic capability based on a whole-genome sequence.

## 2. Materials and Methods

### 2.1. Isolation and Culture Conditions

Strain QXD-8^T^ was isolated from the soil sample of an experimental field belonging to the Institute of Geographic Sciences and Natural Resources Research, the Chinese Academy of Sciences, Heilongjiang Province, China. The soil was collected at the site with a longitude of 123°97′184″ E, a latitude of 47°34′539″ N, and an altitude of 146 m.

The CAM medium, with sodium carboxymethylcellulose as carbon source, was used to isolate the strain. CAM medium components include (per liter): 10 g of sodium carboxymethylcellulose, 0.2 g of MgSO_4_·7H_2_O, 1.2 g of NaNO_3_, 3 g of K_2_HPO_4_, 6 g of NH_4_Cl, 0.05 g of CaCl_2_, 0.01 g of MnSO_4_·7H_2_O, and 0.01 g of ZnSO_4_·7H_2_0. The pH of the medium was adjusted to approximate 7.0 with a 1 M NaOH solution. Approximate 5 g soil samples were added to the CAM liquid medium and shake-flask cultivation was carried out for 7 days at 15 °C. The enriched culture samples were diluted and spread on CAM plates with 2% (*w*/*v*) agar and cultured at 15 °C for 2 weeks until single colonies formed. The colonies were sub-cultured on CAM agar plates using plate streaking to obtain pure cultures. The isolated strain QXD-8^T^ was stored in 20% glycerol at −80 °C.

### 2.2. 16S rRNA Phylogeny

The 16S rRNA gene of strain QXD-8^T^ was obtained by PCR amplification using 27F (5′-AGAGTTTGATCCTGGCTCA-3′) and 1492R (5′-AGAGTTTGATCCTGGCTCAG-3′) universal primers [26]. The sequence was uploaded into the EZBioCloud webserver (https://www.ezbiocloud.net, accessed on 15 October 2022) to find out closely related species. The 16S rRNA gene sequences of the type strains of 34 closely related species were downloaded from EZBioCloud. Multiple sequence alignments were performed using the CLUSTAL W program [27], and phylogenetic trees were constructed based on the 16S rRNA gene in MEGA X (version 10.2 GUI for Windows) [28] software using maximum likelihood (ML) [29], neighbor-joining (NJ) [30], and minimum evolution (ME) methods. Bootstrap analysis was used with 1000 resamples to evaluate the tree topology [31]. 

### 2.3. Genome Features

The draft genome was sequenced using the Nova PE150 platform using MAGIGENE technologies (Guangdong Province), PR China. The raw reads resulting from the genome of strain QXD-8^T^ were checked with metaWRAP (version 1.2.2) [32]. Filtered reads were assembled with the metaWrap-Assembly module and the quality of the assembly was quantified with QUAST (version 5.0.2) [33]. Genome completeness and contamination were estimated with CheckM software (version 1.0.12) [34]. The genome sequence of strain QXD-8^T^ was initially annotated using PGAP (https://www.ncbi.nlm.nih.gov/genome/annotation_prok/, accessed on 1 July 2023). The G + C content was also calculated from the whole-genome sequence.

CAZyme genes were identified in the genomes using dbCAN3 (carbohydrate-active enzyme, https://bcb.unl.edu/dbCAN2/blast.php, accessed on 15 September 2023) [35], and the families with cellulase activity were included. The enzymes’ localization was predicted using SignalP-5.0 [36] (https://services.healthtech.dtu.dk/services/SignalP-5.0/, accessed on 15 September 2023). Transmembrane regions were predicted using DeepTMHMM [37].

### 2.4. Phylogenomic Analysis and Sequence Identity

Strain QXD-8^T^ was identified as a *Microbacterium* by the GTDB-tk [38]. A total of 285 *Microbacterium* genomes were screened from the Genome Taxonomy Database (GTDB, https://gtdb.ecogenomic.org/, accessed on 10 April 2023). All of these genomes are representative genomes in the GTDB. The genome of *Agreia bicolorate* VKM Ac-2052 was selected as an outer clade. At the same time, the GTDB-tk was used to align QXD-8^T^, with the above genome based on 120 conserved proteins. IQTREE was used to reconstruct phylogenetic trees [39]. The phylogenetic tree was visualized with the iTOL website (https://itol.embl.de/, accessed on 15 October 2023) [40]. The genomes of the seven species most closely related to strain QXD-8^T^ were selected from the phylogenetic tree for further analyses. The average nucleotide identity (ANI) and average amino acid identity (AAI) were calculated using ANI/AAI-Matreix in the Kostas lab Tools webserver (http://enve-omics.ce.gatech.edu/, accessed on 5 April 2023). The digital DNA-DNA hybridization (dDDH) values of strain QXD-8^T^ and the other seven strains were obtained by submitting sequence information on a genome-to-genome distance calculator (GGDC) website (https://ggdc.dsmz.de/ggdc.php, accessed on 5 April 2023).

### 2.5. Physiology, Chemotaxonomy, and Morphology

The tryptic soy broth (TSB) medium (produced by Solarbio Life Sciences in Beijing, China) was utilized to analyze the physiological characteristics of strain QXD-8^T^. The TSB medium, with different NaCl concentrations (0–7%, at intervals of 1%, *w*/*v*), was used to test the optimum salinity range for growth purposes. The pH values supporting growth were tested between 5 and 9.5 (at intervals of 0.5 pH unit) and different buffers were used to maintain stable pH values: 1,4-piperazinediethanesulfonic acid (PIPES for pH 5.5–6.7), phosphate-buffered saline (PBS for pH 6.5–8), and N-cyclohexyl taurine (CHES for pH 8.6–10.0). The temperature growth range was determined at 10, 15, 20, 25, 28, 30, 37, and 45 °C. OD_600_ values were measured with a spectrophotometer (TN5000, produced by YOKE INSTRUMENT in Shanghai, China). The analysis of variance was performed using a one-way ANOVA in GraphPad. The activity of catalase was measured by picking a small number of cells into 3% (*v*/*v*) H_2_O_2_. Gram staining was performed using crystal violet and safranin strains to observe the cell color under a light microscope. The biochemical characteristics of the novel strain and the reference strains were tested using API 20NE and API50 CH kits in conjunction with API 50CHB medium kits (produced by Biomerieux, Shanghai, China), in line with the manufacturer’s instructions. The strain was cultured in a TSB medium at 28 °C for two days. Cell morphology was observed using scanning electron microscopy (SEM, Regulus 8100, produced by HITACHI, Tokyo, Japan), and the motility pattern was observed using light microscopy. At the same time, the type species *Microbacterium lacticum* DSM 20427^T^ were used for parallel experiments.

### 2.6. Cellulose Degradation

The sodium carboxymethylcellulose hydrolytic activity of strain QXD-8^T^ was identified using the Congo red staining method [41]. Firstly, 10 µL of bacterial suspension was added to the CAM medium. After culturing at 15 °C and 28 °C for 5 days, the size of the bacterial circle (S) was measured. The medium was stained by adding 5 mL of 0.1% Congo red staining solution for 10 min, and then 5 mL of 1 M NaCl was added for 10 min to decolorize the dye. The diameters of the hydrolysis circle (H) and colony (S) were measured. Finally, the EI value ($\mathrm{EI}=\frac{\mathrm{diameter}\;\mathrm{of}\;\mathrm{hydrolysis}\;\mathrm{zone}}{\mathrm{diameter}\;\mathrm{of}\;\mathrm{colony}}$) was calculated to quantify its hydrolysis ability.

In addition, we used the CAM-M medium by replacing sodium carboxymethyl cellulose in the CAM medium with cellulose powder and combining it with the TSB medium at a ratio of 8:2. Strain QXD-8^T^ was cultivated at 28 °C. After 0 h, 8 h, 24 h, 32 h, 40 h, 48 h, and 72 h of culture, 1 mL of bacterial solution was taken after centrifugation, and the reducing sugar content of the supernatant was determined using a cellulase assay kit (produced by OKA, Beijing, China) based on the dinitrosalicylic acid (DNS) method.

The CAM-C medium was used to determine the growth of strain QXD-8^T^ by replacing sodium carboxymethyl cellulose in the CAM medium with cellobiose and combining it with TSB medium at a ratio of 8:2. The multifunctional microplate reader (EnSpire 2300, produced by FUJITSU, Kawasaki, Japan) was used to determine the absorbance value of strain QXD-8^T^ at 600 nm at different temperatures to determine the growth of the strain. The analysis of variance was performed using one-way ANOVA in GraphPad.

## 3. Results

### 3.1. General Features

**Figure 1:**
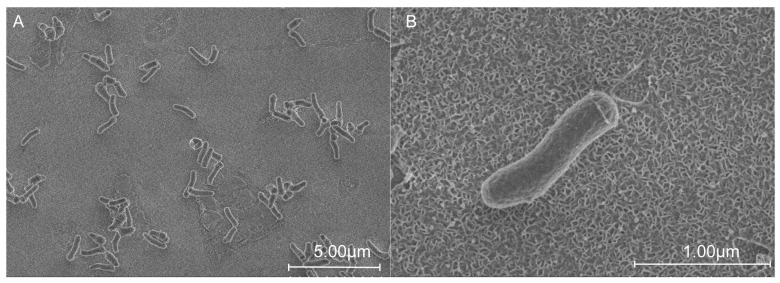
Morphological observation of strain QXD-8^T^ using scanning electron microscopy. The cells were cultured in TSB at 28 °C for 2 days. Magnification levels of ×6.0k (**A**) and ×45.0k (**B**).

The cells of strain QXD-8^T^ were rod-shaped, and varied from 0.5 to 0.7 μm in width and from 1.0 to 2.2 µm in length (Figure 1). After culturing strain QXD-8^T^ with the TSA medium for 2 days, colonies (with a diameter of about 1 mm) formed and they were round, raised, light yellow, and moist. Light microscopy showed that the strain was not motile. Strain QXD-8^T^ was a Gram-stain-positive bacterium.

The draft genome of strain QXD-8^T^ was sequenced using next-generation genome sequencing technology, and was deposited at the NCBI (GCA_031085295.1). The size of the genome was 4.163 Mb. The genomic DNA G + C content was 70.1 mol%. Strain QXD-8^T^ had 1 16S rRNA gene, 1 23S rRNA gene, 1 5S rRNA gene, 47 tRNA genes, and 1 tmRNA (transfer messenger RNA) gene. A total of 4110 coding sequences (CDSs) were predicted to exist in the genome.

### 3.2. Phylogeny and Taxonomy

**Figure 2:**
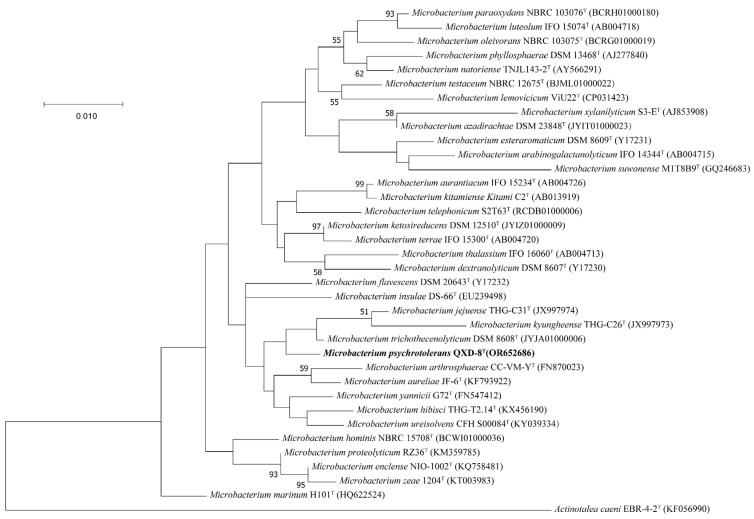
Maximum likelihood phylogenetic tree based on 16S rRNA gene sequences. Bootstrap values (%) were based on 1000 replicates and shown with more than 50% bootstrap support. The sequence of *Actinotalea caeni* EBR-4-2^T^ was used as the outgroup. Bar, 0.010 substitution per nucleotide position. The name of the strain obtained in this study is bold.

The partial 16S rRNA gene sequence (1401 bp) of strain QXD-8^T^ was obtained by PCR amplification, while the whole-length 16S rRNA gene sequence (1520 bp) of strain QXD-8^T^ was obtained from the genome-wide annotation information (described below), and the accession number of the latter is Q9R08_19460 at the NCBI. The two sequences showed 100% identity. The similarity analysis of the whole-length 16S rRNA gene sequence on EZBioCloud website showed that strain QXD-8^T^ had high similarities of 99.10%, 98.82%, and 98.82% with *Microbacterium trichothecenolyticum* DSM 8608^T^, *Microbacterium ketosireducens* DSM 12510^T^, and *Microbacterium yannicii* G72^T^, respectively. The similarities between QXD-8^T^ and other species were lower than 98.7%. Therefore, strain QXD-8^T^ could be considered a member of the genus *Microbacterium*. Phylogenetic trees of 16S rRNA gene sequences were constructed with ML (Figure 2), NJ (Figure S1), and ME methods (Figure S2), revealing that QXD-8^T^ clusters with *Microbacterium trichothecenolyticum* DSM 8608^T^, *Microbacterium jejuense* THG-C31^T^, and *Microbacterium kyungheense* THG-C26^T^ in the genus *Microbacterium*, and forms a separate clade with bootstrap supports of less than 50%, suggesting low credibility. A similar situation was also reported in the phylogenetic analyses of other species [20].

**Figure 3:**
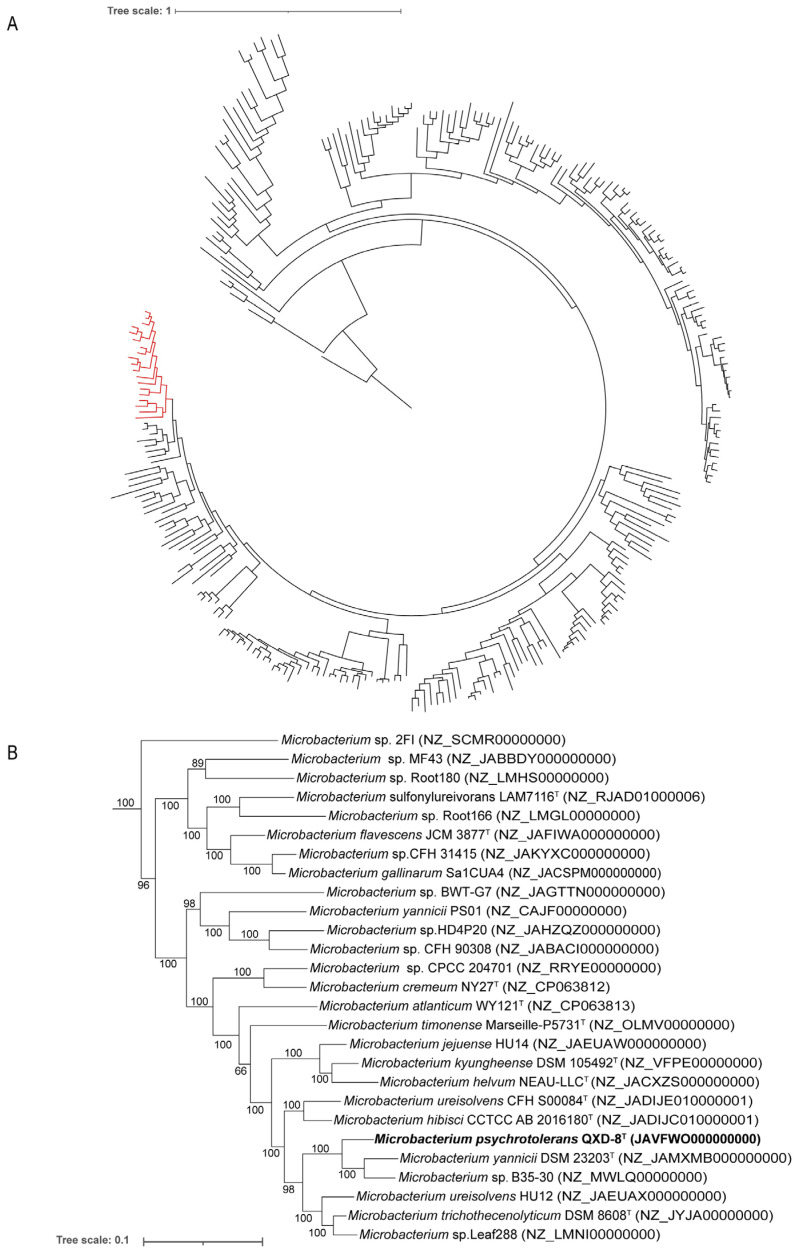
Maximum likelihood phylogenetic tree based on 120 conserved proteins in the genome. (**A**) The phylogenomic tree was composed of 285 *Microbacterium* genomes. Bar, 1 substitution per amino acid position. (**B**) The red part in subfigure A to show the details of the branch including QXD-8^T^. Bar, 0.1 substitution per amino acid position. Bootstrap values (%) were based on 1000 replicates, and more than 50% bootstrap support was exhibited. The name of the strain obtained in this study is bold.

To clearly identify the phylogenetic placement of strain QXD-8^T^, a phylogenomic tree was then constructed based on 120 conserved proteins. The genomes of 285 species in the genus *Microbacterium* were included from the GTDB. Specifically, strain QXD-8^T^ was closely related to *Microbacterium yannicii* G72^T^ and *Microbacterium* sp. B35-30 with almost 100% bootstrap support (Figure 3). Evidently, the strain represents a new taxon within the genus *Microbacterium*.

In order to confirm the representativeness of strain QXD-8^T^ as a new species within the genus *Microbacterium*, similarity analyses at the genome level were performed. The seven validly published species that shared a close relationship with QXD-8^T^ in the phylogenetic analyses, i.e., *Microbacterium lacticum* DSM 20427^T^, *Microbacterium yannicii* G72^T^, *Microbacterium ureisolvens* CFH S00084^T^, *Microbacterium trichothecenolyticum* DSM 8608^T^, *Microbacterium jejuense* THG-C31^T^, *Microbacterium hibisci* THG-T2.14^T^, and *Microbacterium kyungheense* THG-C26^T^, were selected as references. The ANI, AAI, and dDDH values between QXD-8^T^ and these reference strains were 80–88%, 69–89%, and 19.1–54.5%, respectively (Table 1). All these percentages were lower than the thresholds for the species boundaries of ANI 95–96% [42], AAI 95–96% [43], and dDDH 70% [44], respectively. In particular, it exhibited the highest similarities with *Microbacterium yannicii* G72^T^. Consequently, strain QXD-8^T^ was found to be genotypically distinct from any previously described species and should be assigned to a different species.

Taken together, strain QXD-8^T^ could, in principle, be considered a novel species within the genus *Microbacterium* based on the evidence obtained from the phylogenetic and phylogenomic analyses, as well as the ANI, AAI, and dDDH results. 

### 3.3. Physiology and Chemotaxonomy

To describe the characteristics of strain QXD-8^T^ as a type of new species, the complete phenotypic characterization of this strain was carried out and compared with the seven closely related species in the genus *Microbacterium* (Table 2).

**Figure 4:**
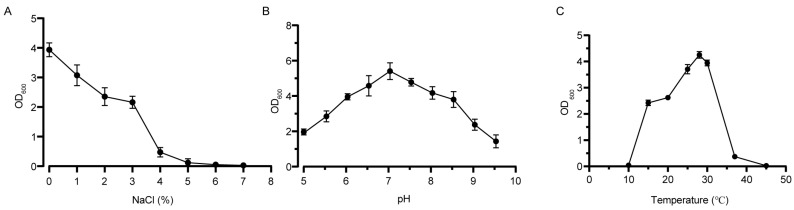
Optical density of the cultivation broth of strain QXD-8^T^ growing under different conditions. The OD600 values of strains at 24 h of growth with different NaCl concentrations (**A**), pH values (**B**), and temperatures (**C**).

Generally, the results showed that strain QXD-8^T^ was able to grow in the NaCl concentration range of 0% to 5%, and grew slowly with an increase in salinity (Figure 4A). Then, a significant difference in strain growth under different salt concentrations was indicated by variance analysis (Figure S3). The pH range for growth was 5.0 to 9.5 (Figure 4B). One-way ANOVA exhibited that there was no significant difference in the growth of strains with different pH levels (Figure S4). The optimum NaCl concentration and pH were 0% and 7.0, respectively. However, it was interesting to find that the strain exhibited similar growth at 15 °C and 20 °C, and its optimum growth temperature was 28 °C. One-way ANOVA also supported this result, demonstrating no significant difference in growth at 15 °C and 20 °C (Figure 4C and Figure S5).

Bubble formation was observed in 3% hydrogen peroxide with a small number of cells, indicating positive peroxidase activity. Other enzyme activities, physiological features, and carbon assimilation results of strain QXD-8^T^ were detected using the API 20NE kit, in line with the manufacturer’s instructions. The results showed that strain QXD-8^T^ was positive for oxidase, nitrate reduction, aesculin hydrolysis, gelatin hydrolysis, *β*-galactosidase, and N-acetylglucosamine hydrolysis. H_2_S production, urease, glucose acidification, indole production, and arginine dihydrolase were negative. Malate, citrate, glucose, arabinose, mannose, and mannitol were utilized as a carbon source. The results of the API 50CH kit showed that acid was produced from L-arabinose, D-xylose, methyl-*β*-D-pyranoxyloside, D-galactose, D-glucose, D-fructose, D-mannose, mannitol, N-acetylglucosamine, arbutin, aesculin iron citrate, salicin, cellobiose, maltose, lactose, sucrose, trehalose, melezitose, raffinose, glycogen, xylitol, gentiobiose, and D-toulon sugar.

According to the results above, strain QXD-8^T^ and related bacteria had a similar carbon source affinity, in terms of an ability to produce acids (Table 2). Compared with the closest related species *Microbacterium yannicii* G72^T^, strain QXD-8^T^ was able to reduce nitrate and used D-lactose and glycogen to produce acid, which were its specific characteristics. In summary, these results provided additional evidence that strain QXD-8^T^ belongs to *Microbacterium* and represents a novel species.

### 3.4. Characteristics of Cellulose Decomposition at Low Temperatures

**Figure 5:**
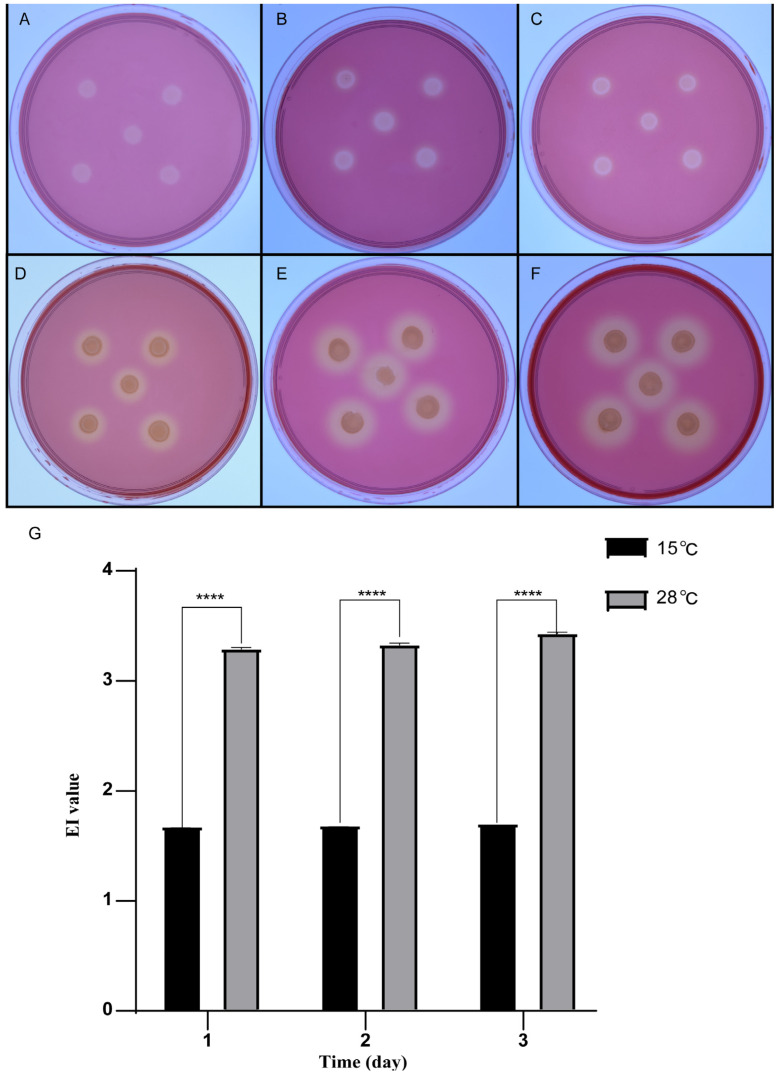
The hydrolysis circle of strain QXD-8^T^ was stained with Congo red on the CAM plate. The cells were incubated at 15 °C for 1 day (**A**), 2 days (**B**), and 3 days (**C**). The cells were incubated at 28 °C for 1 day (**D**), 2 days (**E**), and 3 days (**F**). The graph represents the EI value (diameter of the hydrolysis zone and diameter of the colony) of the transparent hydrolysis zone of strain QXD-8^T^ at 15 °C and 28 °C for different incubation times, and five duplicates were set for each experiment (**G**). All values are representative of five duplicates (****: *p* ≤ 0.0001).

In previous studies, we found that strain QXD-8^T^ was still able to reproduce at low temperatures. Therefore, we performed Congo red staining on strain QXD-8^T^ on the CAM plate to determine whether it could degrade cellulose under low temperatures. The strain was incubated at 28 °C and 15 °C, respectively. It was observed that transparent hydrolysis zones formed from the first day at the two temperatures (Figure 5A,D). Through quantitative analysis, it was found that under the condition of 28 °C, the EI values (the ratio of hydrolysis zone diameter to colony diameter) were 3.29 ± 0.02, 3.33 ± 0.02, and 3.43 ± 0.02 on the first, second, and third days, respectively. Under the condition of 15 °C, the EI values were 1.69 ± 0.02, 1.69 ± 0.02, and 1.71 ± 0.02 on the first, second, and third days, respectively (Figure 5), which were almost half of those at 28 °C. In other words, strain QXD-8^T^ still kept respectable cellulase activity at low temperatures.

**Figure 6:**
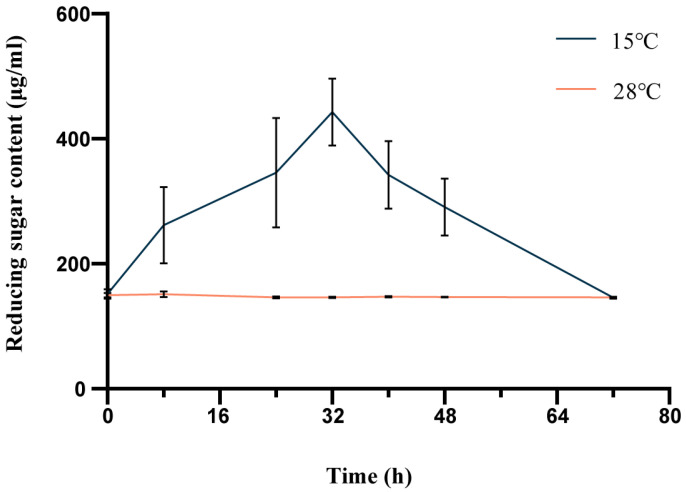
Time course of reducing sugar accumulation levels in the CAM-M medium using strain QXD-8^T^ at 15 °C and 28 °C.

In order to identify the ability of strain QXD-8^T^ to hydrolyze the cellulose, we then used the CAM-M medium (cellulose used as a carbon source) to cultivate the strain and measure the concentration of extracellular reducing sugar. After adding the inoculum, the content of extracellular reducing sugar in the culture medium was measured at the beginning (0 h) as a control. The results showed that under the condition of 15 °C, the extracellular reducing sugar content gradually increased and reached the highest concentration at 32 h. The concentration of reducing sugar was 442.8939 μg/mL, and then gradually decreased to the initial content. However, it was unexpected that the content of reducing sugar was not increased under the condition of 28 °C (Figure 6). The results of one-way ANOVA showed that there were significant differences in some time measurements at 15 °C, while there were no significant differences between the groups at 28 °C (Figures S6 and S7). We speculate that cellulose hydrolysis requires extracellular enzyme activity, and the produced reducing sugars ultimately enter the culture broth, but they can be produced simultaneously through hydrolysis and metabolized by the strain at 28 °C. At low temperatures, due to the slower growth rate, its metabolic activity was significantly weakened. Therefore, reducing sugars can be observed to accumulate first and gradually consume.

**Figure 7:**
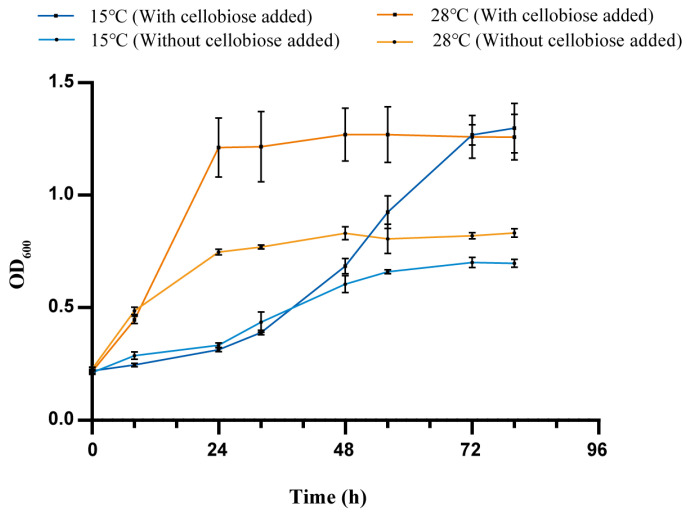
Time course of reducing sugar accumulation and cell growth with cellobiose. Growth curve of strain QXD-8^T^. The growth with and without cellobiose was measured at 15 °C and 28 °C, respectively. The results of one-way ANOVA analyses are exhibited in Figures S8–S11.

Subsequently, in order to dissect the availability of the reducing sugar from the degradation of cellulose using strain QXD-8^T^, we measured the growth of the strain in the CAM-C medium with cellobiose supplemented as a carbon source and without the addition of cellobiose as a control (Figure 7). The results showed that at 28 °C, the group with the addition of cellobiose entered the logarithmic growth phase within 8 h and reached the stationary phase within 24 h, and the final biomass content expressed as OD_600_ could reach about 1.3. However, the growth rate of the strain without cellobiose was lower than that of the strain with cellobiose, and the OD_600_ at the 24th hour of cultivation (at the stationary phase) only reached around 0.75. On the contrary, under the condition of 15 °C, the addition of cellobiose had no obvious effect on the growth of the strain before 48 h. However, after 48 h, the group with added cellobiose entered the logarithmic growth phase and reached the stationary phase within 72 h, indicating that the strain should start to use cellobiose to promote the growth at about 48 h. Overall, strain QXD-8^T^ could decompose cellulose into reducing sugar. It is worth mentioning that the reducing sugar was accumulated under low-temperature conditions.

### 3.5. Genetic Basis of Cellulose Decomposition

**Figure 8:**
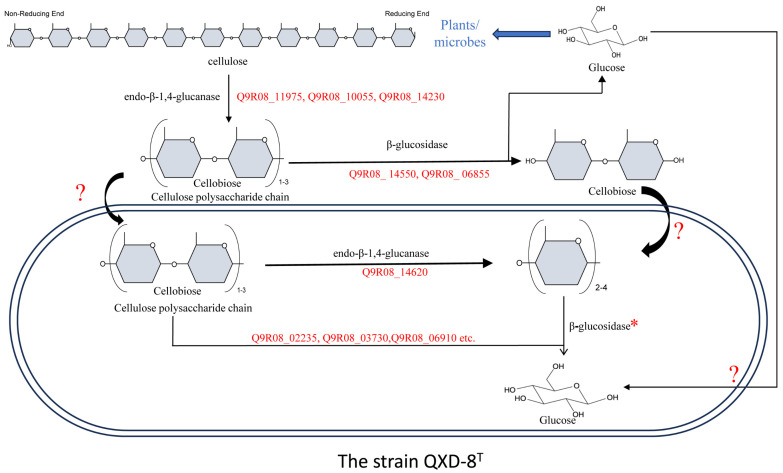
The decomposition process of cellulose using QXD-8^T^. The metabolism pathway was reconstructed based on the genome sequence. Note: The 15 genes encoding intracellular *β*-glucosidase (superscript red star) are Q9R08_02235, Q9R08_03730, Q9R08_06910, Q9R08_16030, Q9R08_16505, Q9R08_11550, Q9R08_03725, Q9R08_13945, Q9R08_14280, Q9R08_15860, Q9R08_19625, Q9R08_01910, Q9R08_09335, Q9R08_01530, and Q9R08_04245. The red question mark: the transport processes of the compounds were not annotated.

In order to understand the cellulose degradation of strain QXD-8^T^, we predicted the genes that encode the enzymes with cellulolytic activity and then reconstructed the pathway through the functional annotation and subcellular localization analyses of the genes in the genome of strain QXD-8^T^. The gene products used for the enzyme families GH5, GH6, and GH10 with endoglucanase activity were annotated (Table 3). The gene Q9R08_14620, assigned as GH5, was not predicted to have signal peptides and transmembrane regions. This protein might play a role in cytoplasm. GH6 exhibited both endoglucanase and cellobiohydrolase activity, and two encoding genes were annotated. The product of gene Q9R08_10050 contained a signal peptide of Tat/SPI, while that of gene Q9R08_14230 contained a signal peptide of Sec/SPI. However, they did not have transmembrane regions and were presumed to function extracellularly. The product of gene Q9R08_11975 was assigned as GH10, and it contained a signal peptide of Sec/SPI type and also had no transmembrane region. It could be an extracellular enzyme. Regarding *β*-glucosidase, the gene of the enzyme families GH1, GH2, GH3, GH39, and GH116 were annotated in this strain (Table 3). Most of them were not predicted to harbor the signal peptide and the transmembrane region. Therefore, they were presumed to play a role in the cell. However, two genes of the GH3 family were predicted to have signal peptides. The product of Q9R08_14550 had a signal peptide of Sec/SPI, while that of Q9R08_06855 had a signal peptide of Sec/SPII (Table 3). Based on the genome sequence, we reconstructed the degradation pathway of cellulose in strain QXD-8^T^. In short, cellulose was found to be cleaved extracellularly by the endoglucanase of the GH6 and GH10 enzyme family to form oligosaccharide chains with reducing and non-reducing ends. A part of the oligosaccharide chains entered the cell and was hydrolyzed by the endoglucanases of the GH5 enzyme family to cellobiose, cellotriose, or cellotetrasose, which were then hydrolyzed to glucose by *β*-glucosidase, and the other part was hydrolyzed to cellobiose or glucose by *β*-glucosidase out of the cell, and cellobiose will continue to be hydrolyzed to glucose. Cellulose was eventually hydrolyzed to glucose or oligosaccharides by the combined action of different enzyme families [50] (Figure 8). The physiological features of cellulose degradation and reducing sugar accumulation supported the metabolism pathway predicted from the genome sequence.

## 4. Discussion

In this study, we isolated a strain QXD-8^T^ that represents a novel species in the genus *Microbacterium* and is capable of degrading cellulose to produce reducing sugar at low temperatures [23,45,46,47,48,49]. Phylogenetic analysis, genomic similarity, chemical classification, and physiological comparison supported the notion that the QXD-8^T^ could be considered the type strain of a new species of the genus *Microbacterium* (Figure 1 and Figure 2, Figures S1 and S2, and Table 2). In the phylogenetic analysis based on the 16S rRNA gene, the topological structure was not highly confident because of low bootstrap values (<50%). In fact, it was also reported to exist in the species of the same genus [17,18,20,22,23,45,47,48,49], possibly because the 16S rRNA gene was so conserved that it was not able to dissect the phylogenetic relationship of some species in the genus *Microbacterium*. With the development of whole-genome sequencing, the phylogenetic relationship could be accurately estimated using the phylogenomic tree based on the concatenated sequences of single-copied conserved proteins, e.g., using the GTDB [51]. In addition, the other sets were also widely adopted, such as UBCG2, another tool used for extracting bacterial core genes and inferring phylogenetic tree [52]. During the experiment, it was found that strain QXD-8^T^ could still grow well at 15 °C, showing the resistance to low temperatures. In previous studies on the close relatives of the species, it was reported that *Microbacterium yannicii* G72^T^ was also isolated from the middle- and high-latitude areas and could also grow under low temperatures [23]. In addition, *Microbacterium jejuense* THG-C31^T^ [47], *Microbacterium suwonense* M1T8B9^T^ [48], *Microbacterium hibisci* THG-T2.14^T^ [49], and *Microbacterium kyungheense* THG-C26^T^ [47] exhibited similar features. In other words, low temperature tolerance may be a common characteristic of strain QXD-8^T^ and closely related species.

The cellulose breakdown capacity of the strain was demonstrated and the metabolism pathway was deduced based on the reconstruction of the genome sequence. Through genome annotation, we found that the enzyme family is related to the cellulose degradation pathway of the strain, and we explained how these enzyme genes function through the prediction of transmembrane regions and signal peptides. Admittedly, the predicted genes need further biochemical and genetic evidence. The reports of related bacteria do not emphasize their advantages for cellulose degradation, but the genome annotation results at least show that *Microbacterium yannicii* G72^T^, *Microbacterium jejuense* THG-C31^T^, *Microbacterium suwonense* M1T8B9^T^, and *Microbacterium hibisci* THG-T2.14^T^ also harbored the genes of GH1, GH2, GH3, GH6, and GH10, as well as other enzyme families (Table S2). Moreover, in the results of the API20NE kit, most of the carbon assimilation results of closely related bacteria were positive for ferric citrate, which also indicated that it had *β*-glucosidase activity. Therefore, cellulase activity may be a common feature in *Microbacterium*. The genus *Microbacterium* could also be a potential microbial resource for cellulose degradation (Table 2). It has been reported in the literature that the GH48 enzyme family is involved in the degradation of cellulose crystalline regions [53], and we did not annotate the encoding gene from the enzyme family in strain QXD-8^T^. It is possible that exoglucanase can only recognize the amorphous region in the cellulose chain for cutting and interact with *β*-glucosidase to generate glucose, but the crystalline regions in cellulose may not be degraded in strain QXD-8^T^.

In this study, reducing sugars could have accumulated in the extracellular environments under low temperatures, and the addition of cellobiose could have promoted the growth of cells. We speculated that the strain could not quickly transport reducing sugars into the intracellular space for growth under low-temperature conditions, although cellulase and transporter activity rates were reported to be inhibited by low temperatures [11,54]. The notion of whether the extracellular reducing sugars were accumulated or not depends on the cellulase and transporter activity rates at low temperatures. In this study, our results could be explained by speculating that the activity of the transporter apparently decreased more than that of cellulase (Figure 6). The accumulated reducing sugars could also be used by other microorganisms or plants. At the same time, in some previous reports, some plant rhizosphere growth-promoting bacteria (PGPR) promote plant growth by dissolving phosphorus [55], dissolving potassium, and fixing nitrogen, but in these cases, reducing sugars such as glucose are needed to provide energy. In addition, cellulose nitrogen-fixing bacteria (CNFB) can accelerate composting and effectively reduce the volume of compost in straw residue composting treatment [56]. Delving into such microorganisms has a positive impact on our knowledge of the sustainable disposal of agricultural waste. 

## 5. Conclusions

In this study, we isolated a new strain from soil, which was designated QXD-8^T^. Using polyphasic taxonomic methods, including phenotypic, physiological, phylogenetic, and biochemical analyses, strain QXD-8^T^ was identified as a member of the genus *Microbacterium*. Phylogenetic analysis based on 16S rRNA gene sequences and 120 conserved proteins from the whole genome revealed that strain QXD-8^T^ formed a separate clade in the genus *Microbacterium*. Genomic relatedness, assessed by ANI, AAI, and dDDH values, further confirmed that QXD-8^T^ differed from its phylogenetically closest species. Overall, these results support the idea that QXD-8^T^ represents a new member of the genus *Microbacterium*.

The *Microbacterium* sp. QXD-8^T^ was able to grow on the CAM plate with sodium carboxymethyl cellulose as a carbon source at 15 °C and form a transparent hydrolysis circle after Congo red staining. In the liquid medium, it effectively degraded cellulose and produced reducing sugars. Functional annotation revealed the presence of encoding genes for the GH5, GH6, and GH10 enzyme families with endoglucanase activity, as well as the GH1, GH3, GH39, and GH116 enzyme families with *β*-glucosidase activity, suggesting their genetic basis for extracellular cellulose degradation. This study expands the diversity of psychrotolerant cellulolytic bacteria and provides a potential microbial resource for straw returning in high-latitude areas with low temperatures.

## 6. Description of *Microbacterium psychrotolerans* sp. nov.

*Microbacterium psychrotolerans* (psy.chro.to’le.rans. Gr. masc. adj. psychros, cold; L. pres. part. tolerans, tolerating; N.L. part. adj. psychrotolerans, cold-tolerating) cells are Gram-stain-positive, non-motile, catalase-positive, oxidase-positive, and rod-shaped, with a width of 0.5–0.7 µm, a length of 1.0–2.2 µm, an optimum growth condition of 28 °C, and a pH of 7.0 with 0% (*w*/*v*) NaCl. Colonies growing on TSA agar were light yellow, round, raised, and moist. In the API 20NE system, cells are positive for the hydrolysis of aesculin, nitrate reduction, and gelatin hydrolysis. Available carbon sources are malate, citrate, glucose, arabinose, mannose, and mannitol, and all other tests are negative. In the API 50CH test, acid is produced from L-arabinose, D-xylose, methyl-*β*-D-pyranoxyloside, D-galactose, D-glucose, D-fructose, D-mannose, mannitol, N-acetylglucosamine, arbutin, aesculin iron citrate, salicin, cellobiose, maltose, lactose, sucrose, trehalose, melezitose, raffinose, glycogen, xylitol, gentiobiose, and D-toulon sugar. Cells are positive for alkaline phosphatase, esterase (C4), esterase lipase (C8), lipase (C14), leucine arylamidase, valine arylamidase, trypsin hydrolysis, acid phosphatase, naphthol-AS-BI-phosphohydrolase, α-galactosidase, *β*-galactosidase, α-glucosidase, N-acetyl-*β*-glucosaminidase, and α-fucosidase, but negative for chymotrypsin hydrolysis, *β*-glucuronidase, glucosidase, and α-mannosidase.

The type strain, QXD-8^T^ (=CGMCC 1.60112), with a G + C content value of 70.1 mol%, was isolated from the soil sample in Heilongjiang Province, China.

The GenBank accession number for the 16S rRNA gene sequences of *Microbacterium psychrotolerans* QXD-8^T^ is OR652686 and that for the complete genome is JAVFWO000000000.

## Figures and Tables

**Table 1:** Genome relatedness based on ANI, AAI, and dDDH values between QXD-8^T^ and close related species of the genus *Microbacterium*.

Close Related Species	ANI (%)	AAI (%)	dDDH (%)
*Microbacterium yannicii* G72^T^	88	89	54.5
*Microbacterium trichothecenolyticum* DSM 8608^T^	85	85	44.8
*Microbacterium ureisolvens* CFH S00084^T^	84	85	43.3
*Microbacterium hibisci* THG-T2.14^T^	84	82	38.7
*Microbacterium jejuense* THG-C31^T^	83	82	36.7
*Microbacterium kyungheense* THG-C26^T^	83	81	34.5
*Microbacterium lacticum* DSM 20427^T^	80	69	19.1

**Table 2:** Physiological characteristics of strain QXD-8^T^ and eight *Microbacterium* species type strains with the highest similarity scores.

Characteristic	1 *	2 *	3	4	5	6	7	8	9
Motility	-	-	-	-	-	-	-	-	-
Colony colour	Y	Y	Y	Y	Y	Y	Y	Y	Y
Production of H_2_S	-	-	+	-	+	-	N/A	N/A	-
Temperature growth range (°C)	15–37	20–37	04–37	15–37	25–30	12–35	10–40	10–40	12–35
Optimum salinity (%)	0	0	0	0	0	1–2	0–3	0–7	0–6
Optimum pH	7	7	7.5	7	8	7	7	7	7
Catalase activity	+	+	+	+	+	+	+	-	+
Activity/assimilation (API 20NE):									
Oxidase	+	+	+	-	-	+	+	-	+
Urease	-	-	-	+	+	-	-	+	+
Nitrate reduction	+	+	-	-	+	+	+	-	+
Gelatin hydrolysis	+	-	+	+	-	-	+	+	-
Aesculin iron citrate	+	+	N/A	+	N/A	+	+	N/A	N/A
N-Acetylglucosamine	+	+	N/A	-	N/A	+	+	N/A	+
Arabinose sugar	+	+	N/A	-	N/A	+	+	N/A	+
Acid production (API 50CH):									
Mannitol	-	-	-	+	N/A	N/A	-	N/A	N/A
L-Arabinose	+	-	+	+	N/A	-	+	-	-
Methyl-*β*-D pyranoxyloside	+	-	N/A	+	N/A	-	-	N/A	+
D-Glucose	+	+	N/A	-	N/A	+	+	-	+
L-Rhamnose	-	-	N/A	+	N/A	N/A	+	+	N/A
*myo*-Inositol	-	-	N/A	-	N/A	-	+	+	-
Mannitol	+	+	N/A	-	N/A	+	+	-	+
Arbutin	w	-	N/A	-	N/A	+	-	N/A	+
D-Lactose	w	+	-	+	N/A	+	-	N/A	-
Sucrose	+	-	+	+	N/A	-	+	N/A	-
Trehalose	w	-	N/A	+	N/A	+	+	+	-
Melezitose	+	-	N/A	+	N/A	-	-	N/A	-
Raffinose	+	-	N/A	+	N/A	-	-	N/A	-
Glycogen	+	+	-	+	N/A	+	+	N/A	+
Xylitol	w	-	N/A	-	N/A	N/A	-	N/A	N/A
Gentiobiose	+	-	N/A	+	N/A	+	-	N/A	-

Strains: 1, strain QXD-8^T^; 2, *Microbacterium lacticum* DSM 20427^T^; 3, *Microbacterium yannicii* G72^T^ [23]; 4, *Microbacterium ureisolvens* CFH S00084^T^ [45]; 5, *Microbacterium trichothecenolyticum* DSM 8608^T^ [46]; 6, *Microbacterium jejuense* THG-C31^T^ [47]; 7, *Microbacterium suwonense* M1T8B9^T^ [48]; 8, *Microbacterium hibisci* THG-T2.14^T^ [49]; and 9, *Microbacterium kyungheense* THG-C26^T^ [47]. +, positive; -, negative; w, weakly positive; Y, yellow; N/A, non-data. * Determined in this study.

**Table 3:** Prediction results of signal peptide and transmembrane region of cellulose hydrolysis related genes in strain QXD-8^T^ (Table S1).

Activity Rates in Family	CAZy Family	Locus Tag (Q9R08_X)	Signal Peptide Prediction	
			Protein Type	Cleavage Site
endo-*β*-1,4-glucanase	GH5	14620	NA	-
	GH6	10055	(Tat/SPI)	47 and 48
		14230	(Sec/SPI)	39 and 40
	GH10	11975	(Sec/SPI)	29 and 30
*β*-glucosidase	GH1	02235	NA	-
		03730	NA	-
		06910	NA	-
		16030	NA	-
		16505	NA	-
	GH2	11550	NA	-
	GH3	03725	NA	-
		13945	NA	-
		14280	NA	-
		14550	(Sec/SPI)	43 and 44
		15860	NA	-
		19625	NA	-
		01910	NA	-
		06855	(Sec/SPII)	22 and 23
		09335	NA	-
	GH39	01530	NA	-
	GH116	04245	NA	-

NA: other protein type, meaning no signal peptide was annotated.

## Data Availability

The 16S rRNA gene sequence for the isolate QXD-8^T^ has been uploaded to NCBI with the accession number of OR652686. The whole genome shotgun project has been deposited at NCBI with the accession number of JAVFWO000000000 (submission ID SUB13746405, BioSample ID SAMN36886429, and BioProject ID PRJNA1003280).

## Supplementary Materials

The following supporting information can be downloaded at: https://www.mdpi.com/article/10.3390/microorganisms12020303/s1. Figure S1: Neighbor-joining (NJ) phylogenetic tree based on 16S rRNA gene sequences; Figure S2: Minimum evolution (ME) phylogenetic tree based on 16S rRNA gene sequences; Figure S3: One-way ANOVA analysis of Figure 4A; Figure S4: One-way ANOVA analysis of Figure 4B; Figure S5: One-way ANOVA analysis of Figure 4C; Figure S6: One-way ANOVA analysis of the extracellular reducing sugar content of strain QXD-8^T^ at 28 °C; Figure S7: One-way ANOVA analysis of the extracellular reducing sugar content of strain QXD-8^T^ at 15 °C; Figure S8: One-way ANOVA analysis for the group without cellobiose added at 15 °C; Figure S9: One-way ANOVA analysis for the group with cellobiose added at 15 °C in Figure 7; Figure S10: One-way ANOVA analysis for the group without cellobiose added at 28 °C in Figure 7; Figure S11: One-way ANOVA analysis for the group with cellobiose added at 28 °C in Figure 7. Table S1: Prediction of signal peptide and transmembrane regions of cellulose hydrolysis-related proteins in strain QXD-8^T^; Table S2: Cellulase prediction in the type strain of different *Microbacterium* species.
