# Supplementary material for: The Isolation and Characterization of a Novel Psychrotolerant Cellulolytic Bacterium, Microbacterium sp. QXD-8T
Source: Microorganisms. 2024 Jan 31;12(2):303. doi: 10.3390/microorganisms12020303 (PMC10892437; doi:10.3390/microorganisms12020303)
Supplement: Supplementary file 1 [file microorganisms-12-00303-s001.zip › microorganisms-2783596-supplementary.pdf]

Supplementary Materials

The isolation and Characterization of a Novel Psychrotolerant Cellulolytic Bacterium, *Microbacterium* sp. QXD-8^T

Peng An[1,2], Changjialian Yang[2,3], Wei Li[1], Dahe Zhao[2*], Hua Xiang[2,3*]

Author affiliations:

[1] College of Life Science, Sichuan Normal University, Chengdu 610101, China

[2] State Key Laboratory of Microbial Resources, Institute of Microbiology, Chinese Academy of Sciences, Beijing 100101, China

[3] University of Chinese Academy of Sciences, Beijing 100049, China

Corresponding authors:

Hua Xiang, xiangh@im.ac.cn; Dahe Zhao, zhaodh@im.ac.cn

Supplementary tables

Table S1. Prediction of signal peptide and transmembrane regions of cellulose hydrolysis-related proteins in strain QXD-8^T

Activities in Family	CAZy family	Locus tag (Q9R08_X)	Signal peptide prediction		Prediction of transmembrane regions	
			Protein type	Cleavage site	Protein types	Region types
endo-*β*-1,4-glucanase	GH5	14620	Other	none	Globular	Inside 1-460
	GH6	10055	**(Tat/SPI)**	47 and 48	Globular+SP	Signal 1-33<br>Outside 34-331
		14230	**(Sec/SPI)**	39 and 40	Globular+SP	Signal 1-40<br>Outside 41-535
	GH10	11975	**(Sec/SPI)**	29 and 30	Globular+SP	Signal 1-29<br>Outside 30-387
β-glucosidase	GH1	02235	Other	none	Globular	Outside 492
		03730	Other	none	Globular+SP	Signal 1-12<br>Outside 13-471
		06910	Other	none	Globular+SP	Signal 1-10<br>Outside 11-458
		16030	Other	none	Globular	Outside 1-404
		16505	Other	none	Globular	Outside 1-384
	GH2	11550	Other	none	Globular	Outside 1-834
	GH3	03725	Other	none	Globular+SP	Signal 1-13<br>Outside 14-786
		13945	Other	none	Globular+SP	Signal 1-15<br>Outside 16-869
		14280	Other	none	Globular	Outside 1-809
		14550	**(Sec/SPI)**	43 and 44	Globular+SP	Signal 1-43<br>Outside 44-752
		15860	Other	none	Globular+SP	Signal 1-10<br>Outside 11-772
		19625	Other	none	Globular+SP	Signal 1-10<br>Outside 11-801
		01910	Other	none	Globular	Outside 1-600
		06855	**(Sec/SPII)**	22 and 23	Globular+SP	Signal 1-22<br>Outside 23-400
		09335	Other	none	Globular+SP	Signal 1-18<br>Outside 19-767
	GH39	01530	Other	none	Globular	Outside 1-543
	GH116	04245	Other	none	Globular	Outside 1-870

Table S2. Cellulase prediction in the type strain of different *Microbacterium* species

Microbacterium hibisci THG-T2.14^T	
**CAZy family**	**locus_tag**
GH6	NZ_JADIJC010000001.1_82
GH10	NZ_JADIJC010000007.1_48
GH1	NZ_JADIJC010000001.1_508
	NZ_JADIJC010000004.1_309
	NZ_JADIJC010000007.1_152
	NZ_JADIJC010000009.1_69
GH2	NZ_JADIJC010000006.1_205
	NZ_JADIJC010000002.1_215
GH39	NZ_JADIJC010000002.1_330
GH116	NZ_JADIJC010000006.1_44

Microbacterium jejuense THG-C31^T	
**CAZy family**	**locus_tag**
GH6	NZ_JAEUAW010000007.1_4
	NZ_JAEUAW010000008.1_3
GH10	NZ_JAEUAW010000009.1_82
GH1	NZ_JAEUAW010000002.1_30
	NZ_JAEUAW010000002.1_30
	NZ_JAEUAW010000004.1_176
	NZ_JAEUAW010000012.1_77
	NZ_JAEUAW010000012.1_84
GH2	NZ_JAEUAW010000001.1_12
	NZ_JAEUAW010000001.1_135
	NZ_JAEUAW010000001.1_37
	NZ_JAEUAW010000002.1_118
	NZ_JAEUAW010000002.1_138
	NZ_JAEUAW010000008.1_43
	NZ_JAEUAW010000012.1_82
	NZ_JAEUAW010000014.1_23
	NZ_JAEUAW010000023.1_6
	NZ_JAEUAW010000001.1_136
	NZ_JAEUAW010000002.1_42
GH3	NZ_JAEUAW010000001.1_31
	NZ_JAEUAW010000004.1_66
	NZ_JAEUAW010000007.1_152
	NZ_JAEUAW010000008.1_12
	NZ_JAEUAW010000010.1_131
	NZ_JAEUAW010000011.1_48
	NZ_JAEUAW010000012.1_99
	NZ_JAEUAW010000020.1_21
	NZ_JAEUAW010000021.1_32
	NZ_JAEUAW010000027.1_6
GH39	NZ_JAEUAW010000001.1_237
GH116	NZ_JAEUAW010000011.1_77

Microbacterium kyungheense THG-C26^T	
**CAZy family**	**locus_tag**
GH6	NZ_VFPE01000003.1_241
	NZ_VFPE01000001.1_361
GH10	NZ_VFPE01000001.1_731
GH1	NZ_VFPE01000002.1_311
	NZ_VFPE01000002.1_852
	NZ_VFPE01000004.1_161
	NZ_VFPE01000001.1_290
	NZ_VFPE01000002.1_100
GH2	NZ_VFPE01000001.1_629
	NZ_VFPE01000003.1_179
	NZ_VFPE01000003.1_205
	NZ_VFPE01000003.1_28
	NZ_VFPE01000005.1_6
	NZ_VFPE01000008.1_27
	NZ_VFPE01000008.1_48
	NZ_VFPE01000003.1_27
	NZ_VFPE01000004.1_173
GH3	NZ_VFPE01000001.1_195
	NZ_VFPE01000001.1_51
	NZ_VFPE01000001.1_563
	NZ_VFPE01000002.1_194
	NZ_VFPE01000002.1_877
	NZ_VFPE01000003.1_185
	NZ_VFPE01000003.1_233
	NZ_VFPE01000004.1_99
	NZ_VFPE01000005.1_24
GH39	NZ_VFPE01000004.1_3
	NZ_VFPE01000007.1_214
GH116	NZ_VFPE01000006.1_14

Microbacterium suwonense M1T8B9^T	
**CAZy family**	**locus_tag**
GH10	NZ_AP027728.1_1647
GH1	NZ_AP027728.1_1668
	NZ_AP027728.1_2285
	NZ_AP027728.1_2590
	NZ_AP027728.1_2754
	NZ_AP027728.1_2907
	NZ_AP027728.1_98
	NZ_AP027728.1_1992
GH2	NZ_AP027728.1_2905
	NZ_AP027728.1_604
	NZ_AP027728.1_2092
	NZ_AP027728.1_2904
GH3	NZ_AP027728.1_2931
	NZ_AP027728.1_367
	NZ_AP027728.1_628
	NZ_AP027728.1_213

Supplementary figures

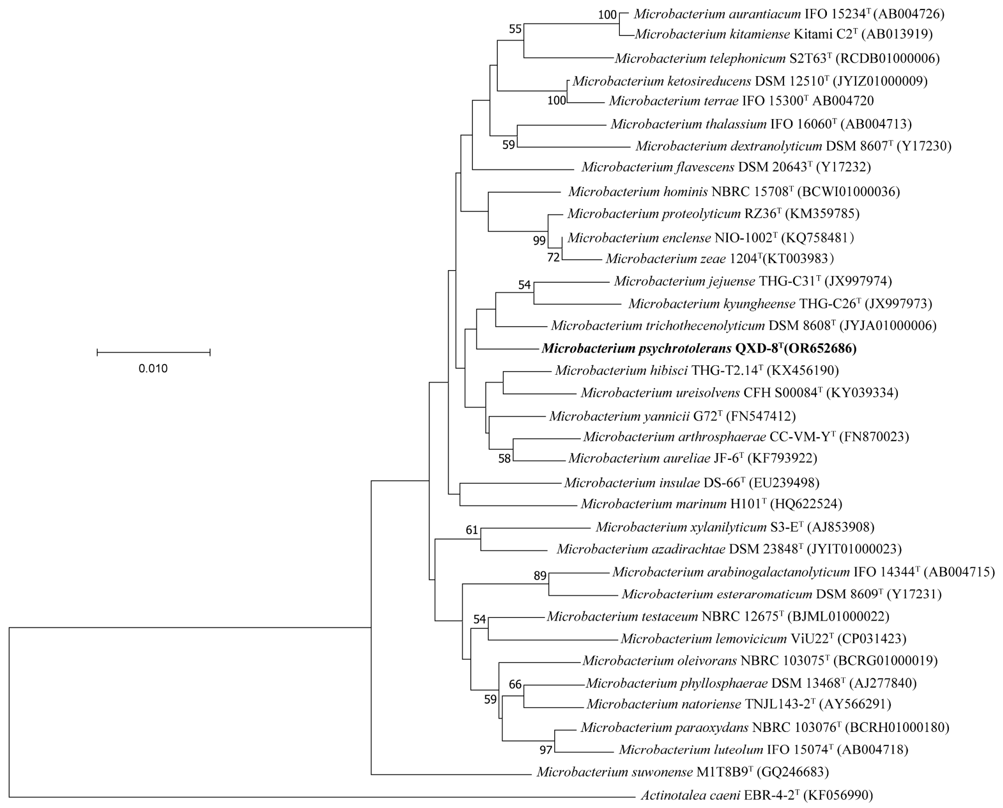

Figure S1. Neighbor-Joining (NJ) phylogenetic tree based on 16S rRNA gene sequences. Bootstrap values (%) were based on 1000 replicates and shown with more than 50% bootstrap support. The sequence of *Actinotalea caeni* EBR-4-2^{T} was used as an outgroup. Bar, 0.010 substitutions per nucleotide position.

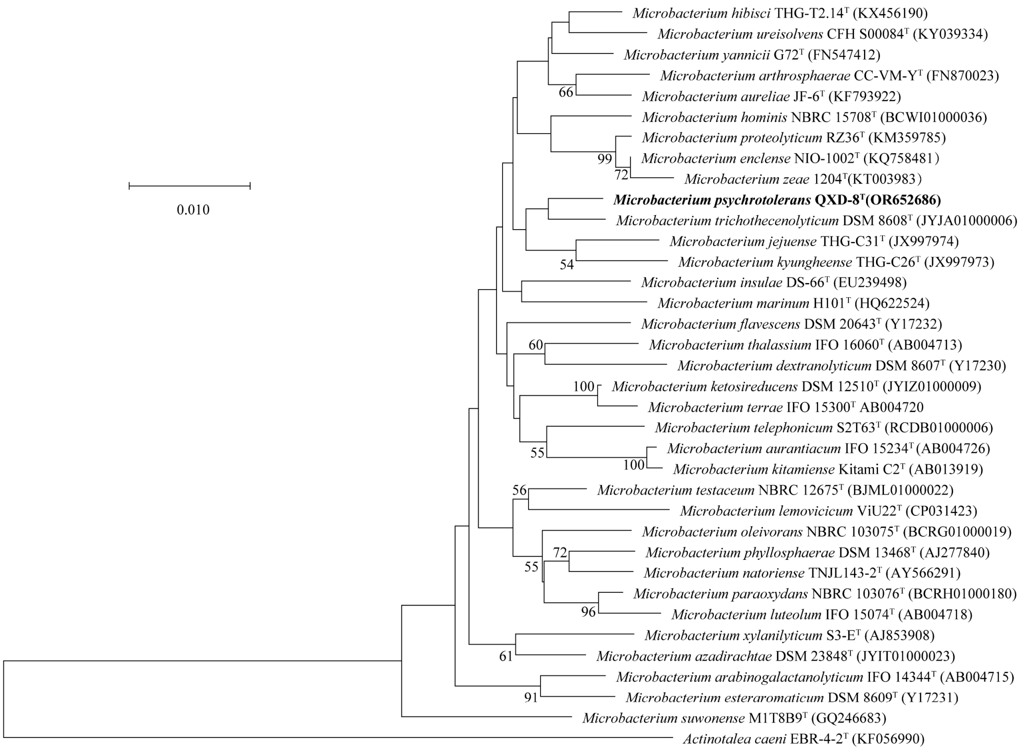

Figure S2. Minimum Evolution (ME) phylogenetic tree based on 16S rRNA gene sequences. Bootstrap values (%) were based on 1000 replicates and shown with more than 50% bootstrap support. The sequence of *Actinotalea caeni* EBR-4-2^T was used as an outgroup. Bar, 0.010 substitutions per nucleotide position.

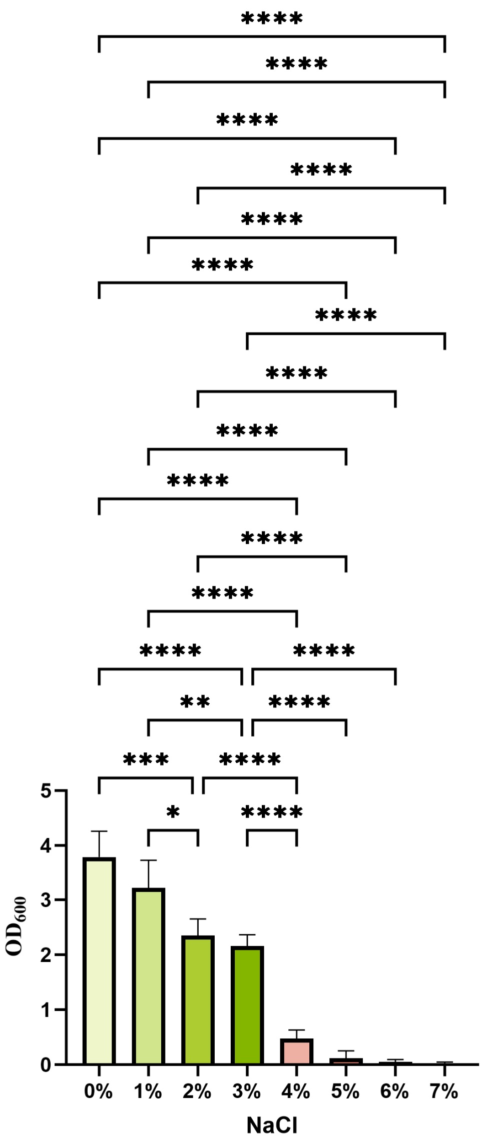

Figure S3. One-way ANOVA analysis of Figure 4A. All values are represented by three duplicates (*: $p \leq 0.05$, **: $p \leq 0.01$, ***: $p \leq 0.001$, ****: $p \leq 0.0001$).

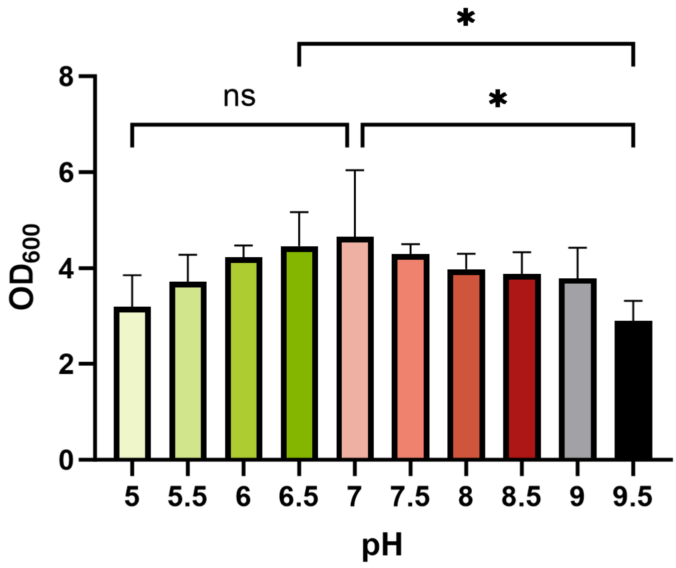

Figure S4. One-way ANOVA analysis of Figure 4B. All values are represented by three duplicates (ns: $p \leq 0.1$, *: $p \leq 0.05$). ns, no significance.

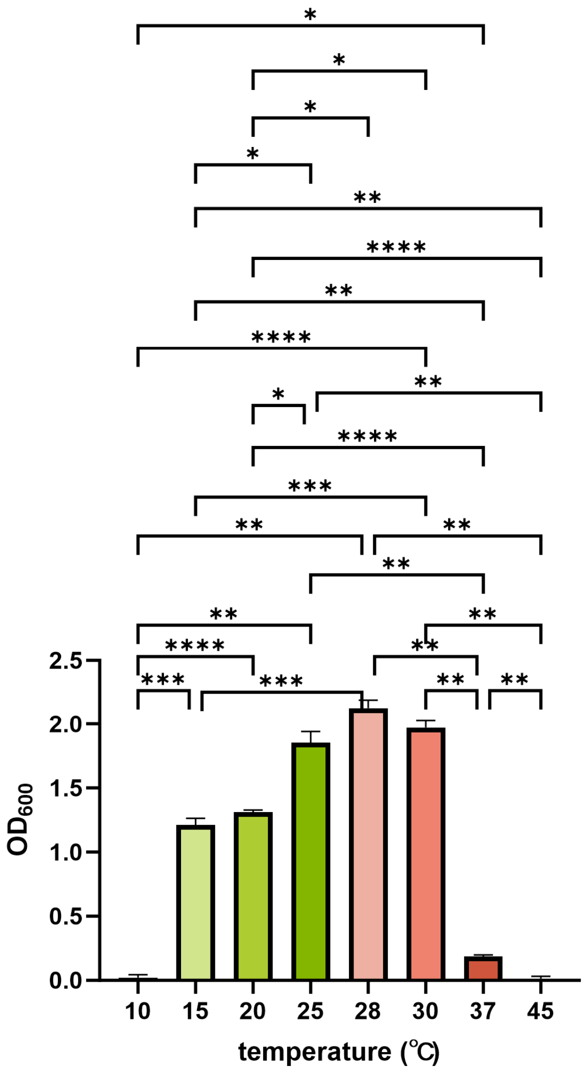

Figure S5. One-way ANOVA analysis of Figure 4C. All values are represented by three duplicates (*: $p \le 0.05$, **: $p \le 0.01$, ***: $p \le 0.001$, ****: $p \le 0.0001$).

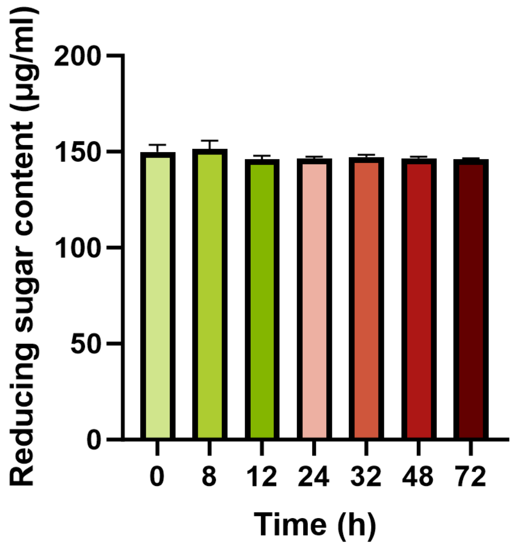

Figure S6. One-way ANOVA analysis of the extracellular reducing sugar content of strain QXD-8^T at 28 °C. The samples were collected at different growth time. All values are represented by three duplicates. No significant difference is present in this experiment.

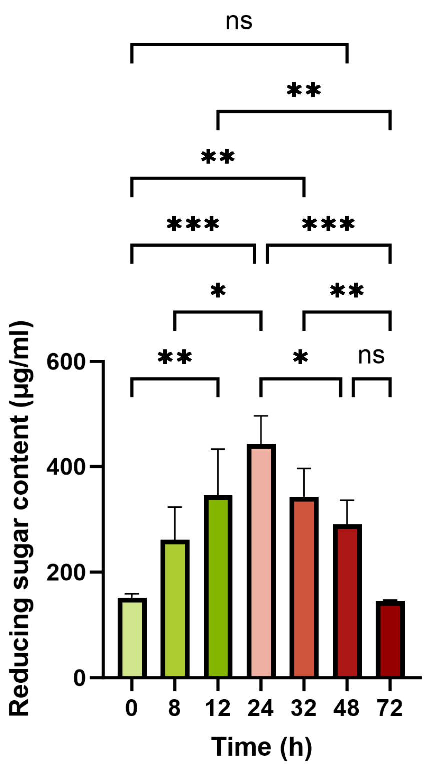

Figure S7. One-way ANOVA analysis of the extracellular reducing sugar content of strain QXD-8^T at 15 °C. The samples were collected at different growth time. All values are represented by three duplicates (ns: $p \leq 0.1$, *: $p \leq 0.05$, **: $p \leq 0.01$, ***: $p \leq 0.001$, ****: $p \leq 0.0001$). ns, no significance.

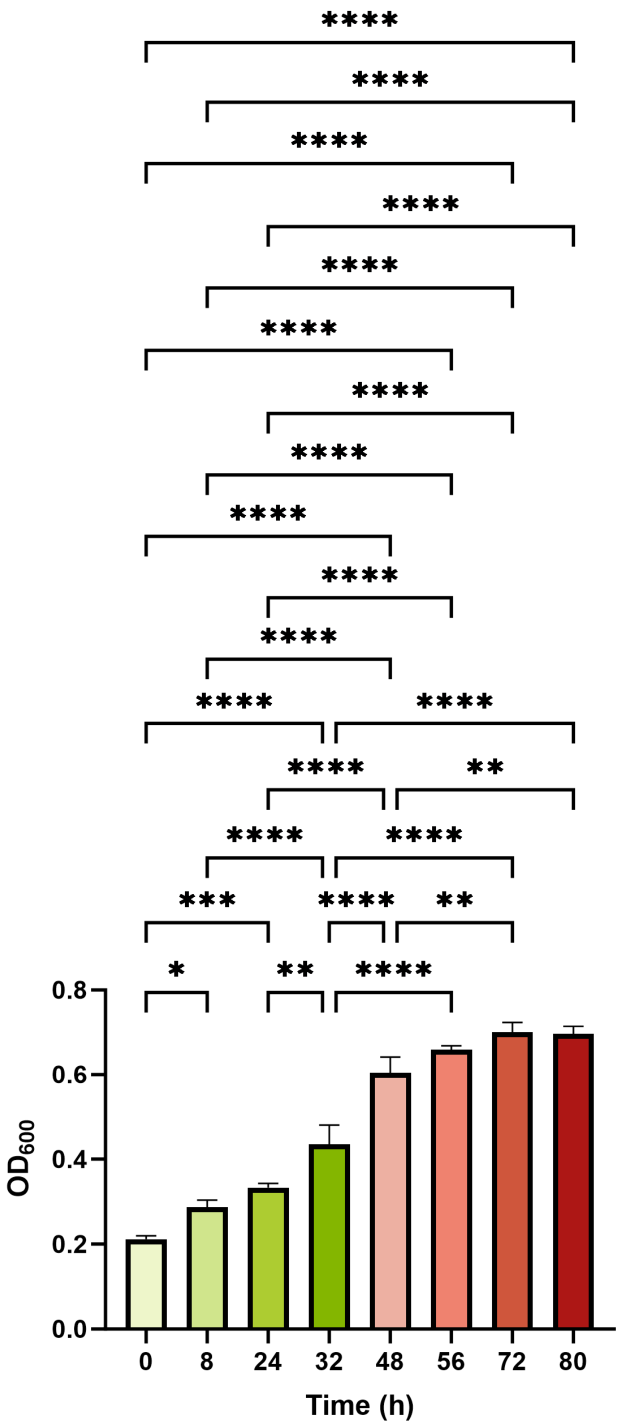

Figure S8. One-way ANOVA analysis for the group without cellobiose added at 15 ℃. All values are represented by three duplicates (***: $p \leq 0.001$, ****: $p \leq 0.0001$).

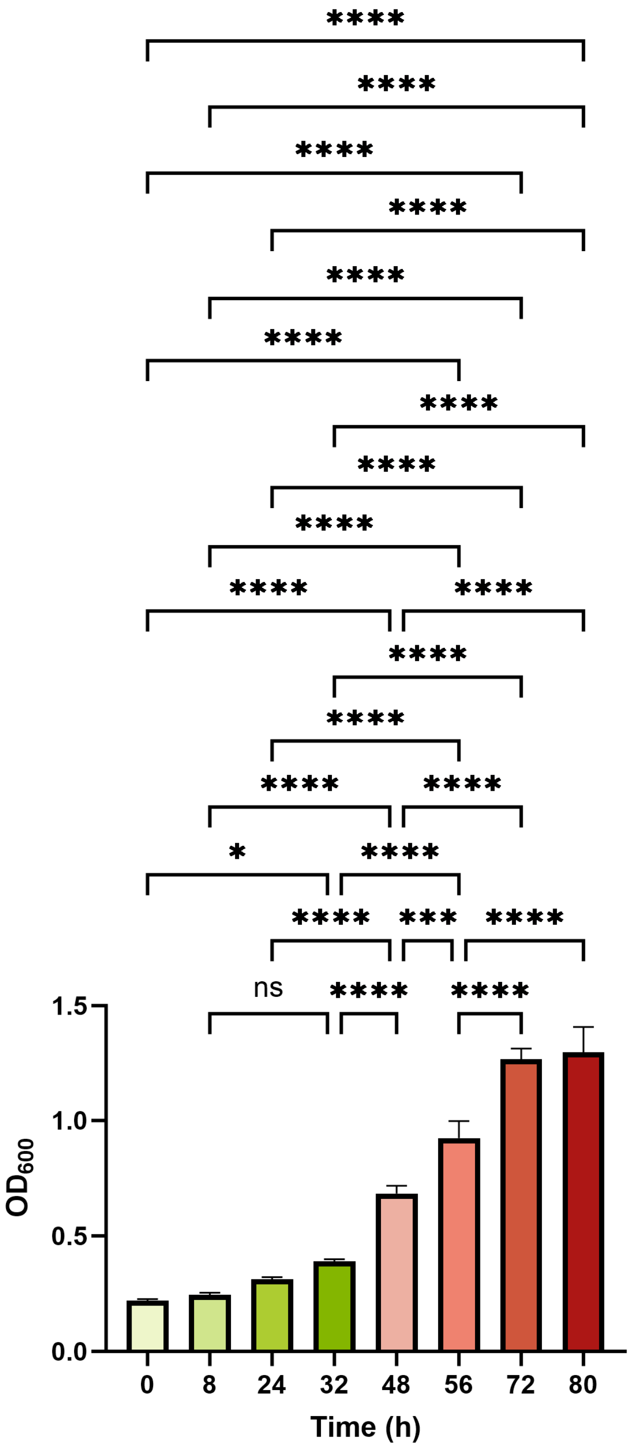

Figure S9. One-way ANOVA analysis for the group with cellobiose added at 15 °C in Figure 7. All values are represented by three duplicates (ns: $p \leq 0.1$, *: $p \leq 0.05$, ***: $p \leq 0.001$, ****: $p \leq 0.0001$). ns, no significance.

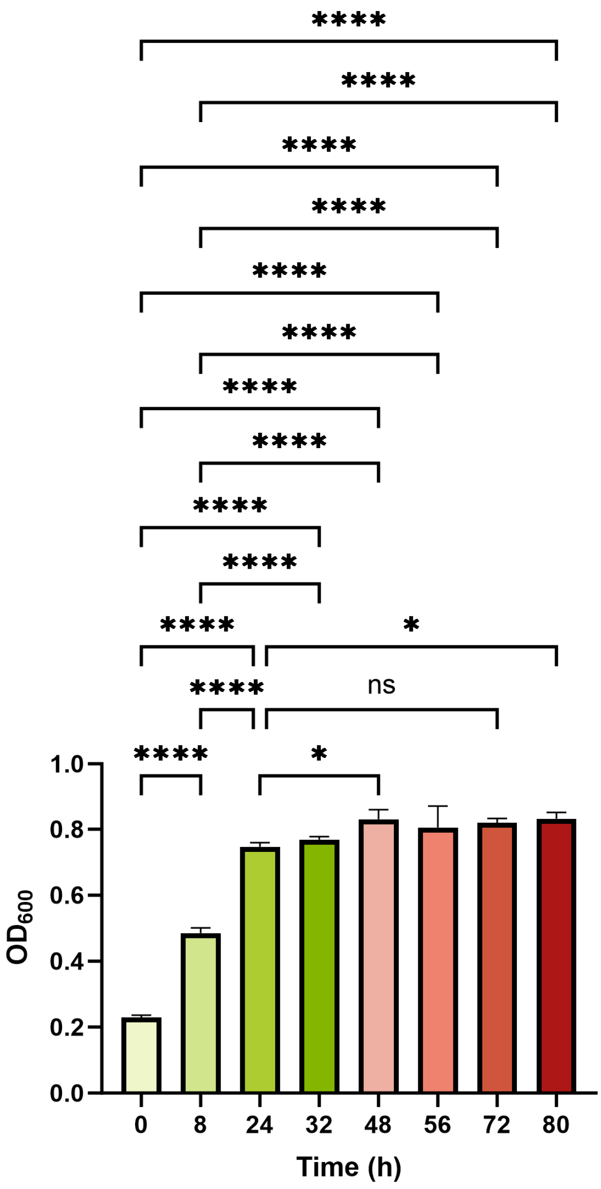

Figure S10. One-way ANOVA analysis for the group without cellobiose added at 28 ℃ in Figure 7. All values are represented by three duplicates (ns: $p \leq 0.1$, *: $p \leq 0.05$, **: $p \leq 0.01$, ***: $p \leq 0.001$, ****: $p \leq 0.0001$). ns, no significance.

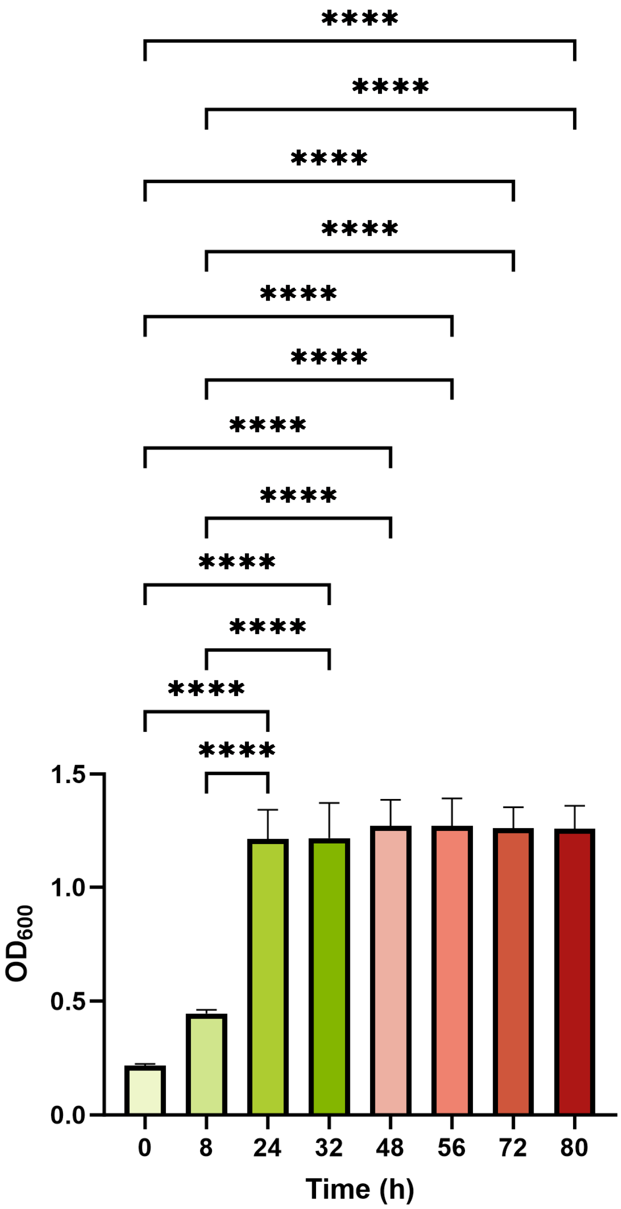

Figure S11. One-way ANOVA analysis for the group with cellobiose added at 28 ℃ in Figure 7. All values are represented by three duplicates (****: $p \leq 0.0001$).
